# Import and transmission of *Mycobacterium orygis* and *Mycobacterium africanum*, Norway

**DOI:** 10.1186/s12879-021-06269-3

**Published:** 2021-06-12

**Authors:** Vegard Eldholm, Janne O. Rønning, Anne Torunn Mengshoel, Trude Arnesen

**Affiliations:** National Reference Laboratory for Mycobacteria, Division of Infection Control and Environmental Health, Norwegian Institute of Public Health, Oslo, Norway

**Keywords:** *Mycobacterium africanum*, *Mycobacterium orygis*, Transmission, Low-incidence country, Whole-genome sequencing

## Abstract

**Background:**

The aim of the current study was to improve our understanding of the origins and transmission of *Mycobacterium africanum* (MAF) in Norway.

**Methods:**

Whole-genome sequences (WGS) were generated for all (*n* = 29) available clinical isolates received at the Norwegian National Reference Laboratory for Mycobacteria (NRL) and identified as MAF in Norway, in the period 2010–2020. Phylogenetic analyses were performed.

**Results:**

The analyses indicated several imports of MAF lineage 6 from both East and West African countries, whereas MAF lineage 5 was restricted to patients with West African connections. We also find evidence for transmission of MAF in Norway. Finally, our analyses revealed that a group of isolates from patients originating in South Asia, identified as MAF by means of a commercial line-probe assay, in fact belonged to *Mycobacterium orygis*.

**Conclusions:**

Most MAF cases in Norway are the result of import, but transmission is occurring within Norway.

## Background

Infections caused by members of the *Mycobacterium tuberculosis* complex (MTBC) other than *M. tuberculosis* make up a significant proportion of tuberculosis cases particularly in West African countries [21]. The incidence of tuberculosis is low in Norway, with less than 400 cases reported per year since 2010. From 2016, the Norwegian National Reference Laboratory for Mycobacteria (NRL) has characterized all MTBC isolates from notified culture positive tuberculosis (TB) cases in Norway, by means of whole-genome sequencing (WGS), significantly improving our ability to both accurately identify species and detect recent transmission. The majority of TB cases in Norway are the result of imported disease rather than transmission in the country [7] Compared to *M. tuberculosis*, *Mycobacterium africanum* (MAF) infections have been suggested to possess reduced capacity for generating active disease and to be less transmissible, but particularly the latter finding is actively debated [1, 11, 13, 17]. The identification by WGS of surprisingly closely related MAF cases, including patients born in countries where MAF is not known to be endemic, prompted us to re-culture, sequence and characterize all MAF isolates received at the NRL from the period 2010–2020.

## Methods

Isolates from the period pre-dating WGS were included based on their assignment to MAF by the GenoType MTBC line-probe assay (Hain). Isolates from 2016 to 2020 were included based on their assignment to the lineages Bov_afri/bov or West African 1 or 2 in our internal pipeline which employs an established tool for lineage definitions [3]. Culturing, DNA extraction and sequencing was performed following methods described earlier [5].

Sequencing reads from all isolates were aligned to H37Rv and SNPs called using the Snippy pipeline (https://github.com/tseemann/snippy) (minfrac 0.9; mapqual 60; basequal 20). SNPs in repeat regions [16] were excluded during generation of the variable-sites multifasta using the ‘snippy-core’ function. Genomes not belonging to West African 1 (Lineage 5) and West African 2 (Lineage 6) based on the initial lineage assignment were searched through the NCBI refseq database using MASH [14] for species identification. A maximum likelihood phylogenetic tree was built using IQ-tree (best model: K3Pu + F + I) with 1000 ultrafast bootstrap replicates.

All sequencing reads and associated metadata is available under European Nucleotide Archive accession PRJEB43202.

Putative transmission clusters were initially identified on the basis of pairwise distances of 12 SNPs or less [18] and further interpreted in light of the patient’s length of residency in Norway.

Tuberculosis is a notifiable disease in Norway. The NRL receives, cultures, stores and performs whole-genome characterization of all successfully cultured MTBC cases in the country. Patient and clinical data is independently reported to the Norwegian Surveillance System for Communicable Diseases (MSIS). Relevant clinical data including country of origin and length of residency in Norway were extracted from MSIS for isolates initially identified as MAF (as described above).

## Results

**Fig. 1:**
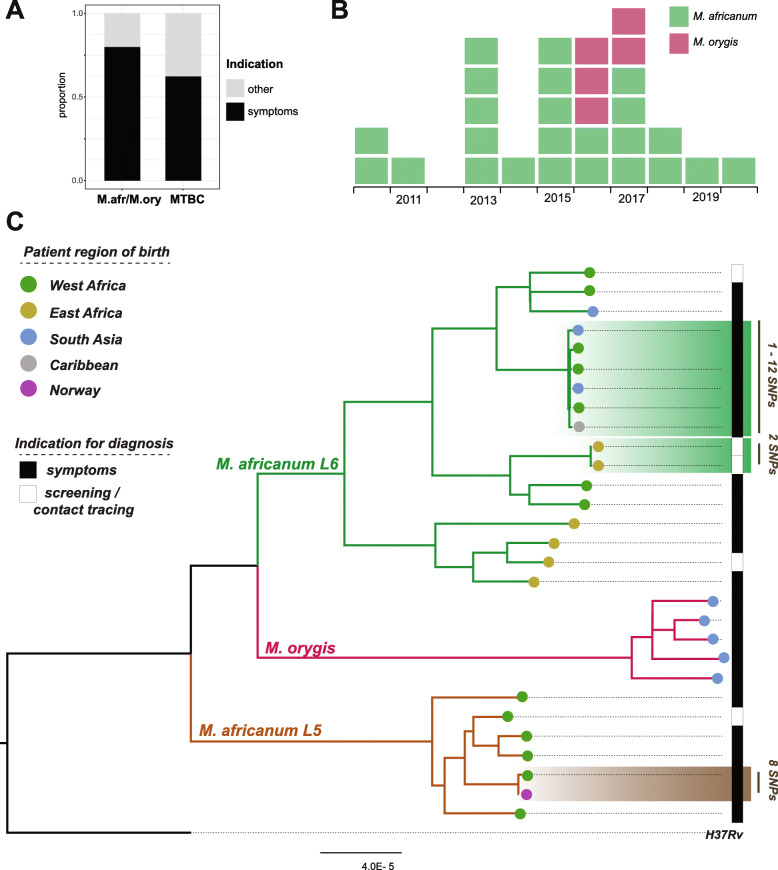
Overview of samples originally identified as *M. africanum* in Norway 2010–2020. (A) Fraction of isolates diagnosed on the basis of symptoms vs all other indications. M.afr/M.ory correspond to the 29 *M. africanum* and *M. orygis* isolates study isolates, whereas MTBC includes all MTBC isolates (*n* = 2818) identified in Norway in the period 2011–2020. (B) Epi-curve illustrating the temporal distribution of the cases. (C) Maximum likelihood phylogeny of the 29 presumed MAF isolates. Clusters compatible with recent transmission are highlighted with boxes colored according to the lineage they belong to

A total of 29 out of 2818 isolates matched the inclusion criteria (see methods). 24 and five out of the 29 cases were diagnosed based on symptoms and contact tracing respectively (Fig. 1A). Closely related isolates were identified in the phylogeny (Fig. 1) and pairwise SNP-distances extracted from the whole genome alignments. The largest cluster contained six isolates, with pairwise SNP-distances of 1–12, suggesting recent transmission by standard criteria [18]. Three of the patients were from countries in Western Africa, where MAF is endemic [10], the other three from South Asia and the Caribbean, where MAF is not known to be present. All six patients resided in the greater Oslo area and four out of six patients had lived in Norway for > 10 years, the remaining two for three to 9 years. The six cases were diagnosed over a 3 year period (2016–2018). Taken together, the available data strongly support the conclusion that the six patients represent a transmission cluster resulting from a single import to Norway.

One pair of MAF isolates separated by eight SNPs were isolated from a West Africa-born person and a Norwegian-born offspring from the same household, likely representing one import event followed by a single transmission event in Norway. In addition, two isolates, both from patients born on the Horn of Africa, were only two SNPs apart, but as the patients had arrived < 6 months prior to diagnosis, it is possible that they had contracted their infection prior to arrival. MAF is not common in the Horn of Africa, but has been reported in genotyping studies [20].

Summarizing the above, we conclude that at least six, possibly seven of the MAF cases were the result of transmission in Norway. Another interesting observation is the clade containing four lineage 6 (L6) cases diagnosed in patients from the Horn of Africa (“East Africa” in Fig. 1). The large genetic distance between the isolates rule out recent transmission, and points to the possibility of four independent imports of MAF L6 from Somalia.

For five patients, all diagnosed after developing symptomatic TB, the infection was found to be caused by *Mycobacterium orygis*. Of the five cases, three were pulmonary and two were extrapulmonary. The isolates had either been erroneously identified as MAF by line-probe assay, or identified as Bov_afri/bov in the SNP-based WGS scheme (TB-profiler). PhyResSE [6] identified the isolates as “Clade 2” (animal lineages and africanum) whereas Mykrobe v.0.9.0 [2] identified the isolates as MAF (species) animal clade A3 (lineage). The apparent lack of accuracy and consistency in these calls prompted us to search for the best matches in the NCBI refseq database (see methods). These searches identified *M. orygis* as the best match for all five isolates. A comparative summary of species and lineage assignments based on the different tools can be found in Table 1.

**Table 1:** Comparative summary of species and lineage assignments from various tools and methods

*MTBC line-probe*	*PhyResSE*	*Mykrobe*	*TB-profiler*	*MASH*
MAF	MAF West African 1a or 1b	MAF Lineage 5	MAF Lineage 5	MAF
MAF	MAF West African 2	MAF Lineage 6	MAF Lineage 6	MAF
MAF	“Clade 2”	MAF animal clade A3	Bov_afri/bov	*M. orygis*

All five patients had been born in South Asia. Two had arrived within 6 months prior to diagnosis, whereas three had lived in Norway for at least 10 years (but could possibly have been latently infected upon arrival or contracted the infection during travels to their countries of birth). The pairwise distances between the five samples were significant, suggesting that all represented unique imports to Norway (Fig. 1).

## Discussion

A study from the USA covering the period 2004–2013 found that MAF exhibited similar clinical characteristics to *M. tuberculosis*, but was suggested to be less transmissible, based on 24-locus mycobacterial interspersed repetitive unit genotyping [17]. The small number of MAF cases in Norway renders a formal assessment of transmissibility futile. However, we find that at least 25% of MAF cases in Norway in the period 2010–2020 were the result of recent transmission (six or seven out of 24 total MAF cases). In line with the study from the USA [17], which found a strong association between tuberculosis caused by MAF and being born in West Africa, 13 out of 24 MAF cases in Norway were diagnosed in people born in West African countries. Our findings adds to the ongoing discussion of the relative transmissibility of MAF compared to *M. tuberculosis* [1, 11, 13, 17], and underscores that MAF infections in low-incidence countries may well be the result of recent local transmission, which is in line with findings from Spain [9].

Mislabelling of *M. orygis* as MAF by the GenoType MTBC line-probe assay (Hain) has been reported earlier [8, 12]. The shortcomings of the above methods in separating *M. orygis* from MAF is likely a result of the close phylogenetic relationship between the two species. Nevertheless, as *M. orygis* forms a monophyletic clade in the phylogeny, identifying species-specific SNPs for accurate species identification from genome sequence data should be feasible.

In South Asia, *M orygis* is a causative agent of tuberculosis in cows, rhesus monkeys and humans [8]. In India specifically, *M. orygis* was indeed found to be more common agent of tuberculosis than *M. bovis* in a recent study [4]. In the current study, *M. orygis* was identified exclusively in patients originating in South Asia, supporting that the species is not an entirely uncommon cause of tuberculosis in the region [4]. No transmission of *M. orygis* was found to have occurred in Norway in the period.

MAF L5 isolates were exclusively isolated from patients born in West Africa, except for one case in a household member born in Norway. The geographic origins of patients infected with MAF L6 were much more diverse, which to some degree reflects a single transmission cluster affecting immigrants from different countries. However, we also note a clade containing four MAF L6 isolates, all isolated from East african patients (Fig. 1). The wider geographic distribution of L6 relative to L5 is in line with earlier findings [10, 15]. From the phylogeny, it seems likely that these isolates represent four unique imports to Norway from East Africa, indicating that MAF L6 might circulate in the region. However, the patients in question could possibly have contracted the infection in immigration centres, refugee camps or similar, as has been observed previously [19].

In MSIS, the indication for test, i.e. screening, contact tracing or symptoms, is registered. Compared to TB cases caused by *M. tuberculosis*, a larger portion of cases caused by MAF and *M. orygis* were tested because of symptoms. This finding might indicate that a notion of MAF being less transmissible resulted in less intense contact tracing around some MAF cases, but could also be a stochastic effect.

## Conclusions

Infections with MAF and *M. orygis* are uncommon in Norway. Lineage 5 isolates were almost exclusively diagnosed in patients originating in West Africa, whereas Lineage 6 infections were diagnosed in patients of more diverse origins. In total, in the study period 2010–2020, about a quarter of MAF cases were inferred to be the result of transmission in Norway. Conversely, all *M. orygis* cases were inferred to be the result of infections independently acquired in South Asia.

In line with earlier findings, we find that both classical and whole-genome typing-methods currently struggle with the separation of *M. orygis* and MAF, reflecting the close phylogenetic relationship between the species as defined. However, as *M. orygis* forms a monophyletic clade in phylogenetic reconstructions, identifying species-specific SNPs for typing schemes should be feasible.

## Data Availability

Short read illumina data is publicly available under European Nucleotide Archive accession PRJEB43202. Metadata including species, lineage and date of isolation is available together with the sequences under the accession.
